# Abnormal fiber end migration in Royal College of Surgeons rats during posterior subcapsular cataract formation

**Published:** 2010-07-31

**Authors:** Anita Joy, Tabraiz A. Mohammed, Kristin J. Al-Ghoul

**Affiliations:** 1 Department of Anatomy & Cell Biology, Rush University Medical Center, Chicago, IL; 2 Department of Ophthalmology, Rush University Medical Center, Chicago, IL

## Abstract

**Purpose:**

Prior structural studies of posterior subcapsular cataract (PSC) development in Royal College of Surgeons (RCS) rats suggest that migration of basal fiber ends was disrupted, ultimately resulting in a PSC. Therefore the goal of this study was to assess the overall migration patterns as well as changes to the structure and cytoskeleton of basal fiber ends during PSC development.

**Methods:**

Lenses from 48 RCS dystrophic rats (RCS/Lav) and 24 genetically matched control animals (RCS-rdy^+^/Lav) from 2 to 8 weeks old were examined. Equatorial diameters were measured and suture patterns were photographed immediately following enucleation/dissection. Right eye lenses were fixed and processed to visualize the actin cytoskeleton via laser scanning confocal microcopy (LSCM), left eye lenses were decapsulated, fixed and processed for scanning electron microscopy (SEM). Scaled 3D-computer assisted drawings (CADs) and animations were constructed from the data to depict the changes in suture patterns and fiber end architecture.

**Results:**

At 2 weeks, dystrophic lenses displayed an inverted Y suture on the posterior, and by 3 weeks most lenses had at least one sub-branch. Additional sub-branches were observed with time, opacities being visible as early as 4 weeks and progressing into PSC plaques by 6 weeks. Control lenses displayed inverted Y sutures at all ages and were transparent. SEM of dystrophic lenses revealed fiber ends with normal size, shape, arrangement, and filopodia at 2 weeks; scattered areas of dome-shaped fiber ends and small filopodia were present at 3 weeks. At 4 weeks the irregularly arranged domed fiber ends had extremely long filopodia with ‘boutons’ at their tips. By 6 weeks all fiber ends within plaques displayed rounded or domed basal membranes and lacked filopodial extensions. Control lenses at all time points had comparable ultrastructure to the 2 week old dystrophic lenses. F-actin arrangement within the basal membrane complex (BMC) of control lenses showed the expected peripheral pattern of labeling at all ages. Dystrophic RCS lenses at 2 weeks were comparable to controls, however by 3–4 weeks they displayed scattered foci of F-actin within the BMC. At all time points thereafter, F-actin was rearranged into a ‘rosette’ pattern of prominent foci at cell vertices.

**Conclusions:**

The data are consistent with the hypothesis that migration of basal fiber ends is altered in a two stage process wherein initially, migration patterns undergo a rapid shift resulting in abnormal suture sub-branch formation. Subsequent cytological alterations are consistent with an eventual cessation of migration, precluding proper targeting of basal ends to their sutural destinations and leading to cataract plaque formation.

## Introduction

Congenital retinal dystrophy in the Royal College of Surgeons (RCS) rat is a recessively inherited anomaly that results in the progressive degeneration of photoreceptor cells in the retina during the later stages of retinal development (third week postnatal) [1-3]. One of the many deleterious effects of this disease includes the formation of a secondary cataract, specifically a posterior subcapsular cataract (PSC). Although the cataract was the first feature noted and described in the RCS rat [4], this animal model has been extensively used to study retinal degeneration. Since the first description of the genetic abnormality responsible for the retinal dystrophy by Bourne et al. [2,5], the early initiation, rapid progression, and the resultant PSC formation in these animals have also been well documented. Similarities between the rodent disease and retinitis pigmentosa in humans have been established [5-8], and the RCS rat is therefore an excellent model to study both autosomal recessive retinitis pigmentosa (ARRP) and PSCs.

In the retina, the first damage occurs approximately 12 days postnatal in the rod outer segments and by 18 days postnatal, the outer segment layer is twice as thick when compared to normal. There is also the presence of outer segment debris within this thickened layer. At about 4 weeks postnatal, this layer reaches its maximum thickness and both the inner segments as well as the photoreceptor nuclei begin to degenerate. By about 40 days postnatal, both inner and outer rod segments have completely disintegrated [3,9-11]. The temporal sequence of events that mark the maximal accumulation of outer segment debris and retinal breakdown closely mirror the changes that occur at the posterior surface of the lens. Studies have found that cataracts in RCS rats may be initiated by toxic lipid peroxidation products that are released during rod outer segment degeneration, and which accumulate in the vitreous [12,13]. Between 4 and 6 weeks postnatal, abnormal fiber growth at posterior ends results in a PSC. In particular, the PSCs exhibit enlarged basal fiber ends that arc away from the suture, toward the vitreous [14,15]. The location and presentation of the structural defects suggest that the process of fiber end migration is disrupted. Furthermore, temporal correlation of these changes reveals that the presumed aberrant fiber end migration commences when retinal degeneration is at its peak. Once this period of dystrophic alteration is past, the lens recovers and lens fibers in subsequent growth shells demonstrate relatively normal end migration to form new posterior sutures. Recovery of the lens results in the initial PSC becoming internalized [15,16]. The present study aims to document the altered migration dynamics at basal fiber ends that lead to both gross and ultrastructural modifications in RCS rat lenses. Our results have revealed that PSC formation is characterized by a defined sequence of morphological changes at the posterior fiber ends beginning as early as three weeks postnatal.

## Methods

### Animals

RCS rat breeding pairs (RCS/Lav=inbred, pink-eyed, dystrophic and RCS-rdy^+^/Lav=genetically matched controls) were acquired courtesy of Dr. M. LaVail, Retinal Degeneration Rat Model Resource (RDRMR), University of California, San Francisco, CA. Animals were housed in the Comparative Research Center of Rush University Medical Center (RUMC), Chicago, IL, and bred as per the schemata laid out by the RDRMR for breeding RCS strains. The animals were cared for in accordance with the ARVO Statement for the Use of Animals in Ophthalmic and Vision Research and all experiments were performed under aseptic and sterile conditions as approved by the Institutional Care and Use Committee (IACUC) of RUMC, Chicago, IL. The RCS experimental animals were randomized into six groups (2, 3, 4, 5, 6, and 7–8 weeks) and genetically matched controls were randomized into three groups (2–3 weeks, 4–6 weeks, and 7–8 weeks). Each group had eight animals with n=72. At the specific age points, animals were euthanized using an intra-peritoneal dose of sodium pentobarbital (1 ml @398 mg/ml).

### Lenses

Immediately following euthanasia, the eyes were enucleated and lenses were dissected under a Stereoscopic Zoom Microscope (Nikon SMZ1500; Nikon Inc., Melville, NY). The equatorial diameter of all lenses was measured and lenses were photographed using a digital camera (Nikon DIX; Nikon Inc., Melville, NY) operated by the Q Capture Pro Software (QImaging Corporation, Surrey, Canada) on a Pentium PC platform. All right eye (OD) lenses were processed for cytochemistry and all left eye (OS) lenses were processed for visualization via scanning electron microscopy (SEM). Statistical analysis was performed to determine significant changes in lens equatorial diameter.

### Laser scanning confocal microscopy (LSCM)

All OD lenses were fixed in 3% paraformaldehyde in 0.07 M phosphate buffered saline (PBS) for 2 h. Fixed lenses were secured on specimen mounting blocks posterior side-up using cyanoacrylate adhesive, embedded in 2.5% agarose gel and sectioned at 100 μm thickness in a vibrating knife microtome (Vibratome Series 3000 Plus-Tissue Sectioning System; Leica Microsystems, Bannockburn, IL). Sections were fixed for an additional 30 min in 3% paraformaldehyde, permeabilized with 0.2% Triton X-100 for 30 min and labeled for F-actin using Phalloidin-TRITC at 1:50 dilution (Sigma, Saint Louis, MO). Labeled sections were mounted on glass coverslips (Fisherbrand, Fisher Scientific, Pittsburgh, PA) with Vectashield mounting medium (Vector Laboratories Inc., Burlington, CA) to prevent photobleaching. The coverslips were sealed to glass slides using a commercially available lacquer.

### Scanning electron microscopy (SEM)

All OS lenses were decapsulated from the anterior aspect to expose the posterior fiber ends. Decapsulated lenses were fixed in 2.5% glutaraldehyde fixative in 0.07 M sodium cacodylate buffer for 3 days with fresh fixative changes daily. Following fixation, lenses were washed in 0.1 M sodium cacodylate buffer, post-fixed in 1% aqueous osmium tetroxide at 4 °C, washed in buffer and dehydrated through a graded ethanol series. Following dehydration with 100% ethanol, the lenses were passed through a graded ethanol/Freon 113 series, critical-point dried in 100% Freon 23 (DuPont, Wilmington, DE) in a Balzers Critical Point Drier 020 (Balzers, Hudson, NH), secured on aluminum stubs with colloidal silver adhesive, sputter coated with gold (Pelco Model 3 Sputter Coater 91000; Ted Pella, Inc., Redding, CA) and examined in a scanning electron microscope (Hitachi S-3000N; Hitachi America Ltd., Brisbane, CA). Digital electron micrographic magnification series were acquired from 50×-4000× for each specimen.

### Scaled 3D-CAD drawings and animations

Whole lens measurements, in conjunction with data from gross lens photographs and Confocal/SEM micrographs were used to create scaled 3 Dimensional-Computer Assisted Design (3D-CAD) drawings and animated models using Corel DRAW Graphics Suite (Corel Corporation, Ontario, Canada) on a Pentium PC platform. These explain and depict the temporal sequence of changes to suture patterns, transparency and basal end architecture in the RCS rat lens during the development of PSCs.

## Results

### Lens size

**Figure 1:**
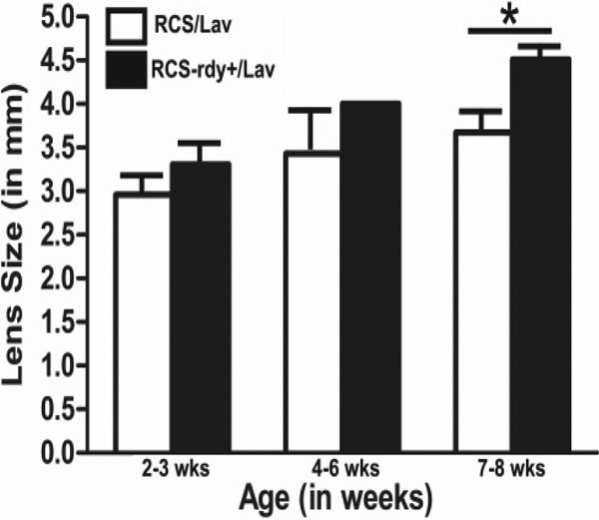
Graphical representation of increase in lens equatorial diameter with age in the dystrophic RCS/Lav lenses and control RCS-rdy^+^/Lav lenses. Lens size increased with age and there was a significant difference in equatorial diameter between the dystrophic and control lenses at 7–8 weeks postnatal (p<0.05).

The RCS/Lav rat lenses gradually increased in size (equatorial diameter) with age from an average of 2.96 mm at 2–3 weeks of age to 3.43 mm at 4–6 weeks of age and 3.76 mm by 7–8 weeks post natal, while the control RCS-rdy^+^/Lav lenses showed an average increase from 3.30 mm at 2–3 weeks, 4 mm at 4–6 weeks, and 4.51 mm at 7–8 weeks of age (Figure 1). In general, the control lenses were larger than the dystrophic lenses and after 4–6 weeks postnatal there was a significant pause in lens growth (equatorial diameter) in the RCS/Lav lenses. Statistical comparison showed that at 7–8 weeks, the dystrophic lenses were significantly smaller than the controls (p<0.05).

### Lens suture pattern and transparency

**Figure 2:**
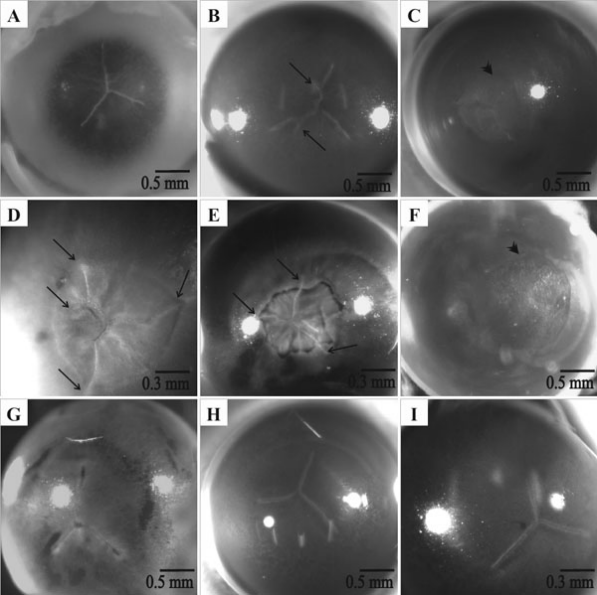
Dissecting microscope photographs of unfixed dystrophic (**A**-**F**) and control (**G**-**I**) RCS rat lenses. **A**: Prior to PSC development, lenses from 2 week old animals displayed three-branched inverted-Y sutures on the posterior surface. **B**: By 4 weeks, one or more abnormal suture sub-branches was apparent (arrows). **C**, **D**: Opacities which circumscribed the suture branches were detected as early as 5 weeks post natal. **E**: The opacity continued to grow through 6 weeks of age. **F**: The PSC completely obscured the suture by 7–8 weeks postnatal. In contrast, the control lenses showed no change from the normal inverted Y suture with age (**G**: 2–3 weeks, H: 4–6 weeks, **I**: 7–8 weeks).

**Figure 3:**
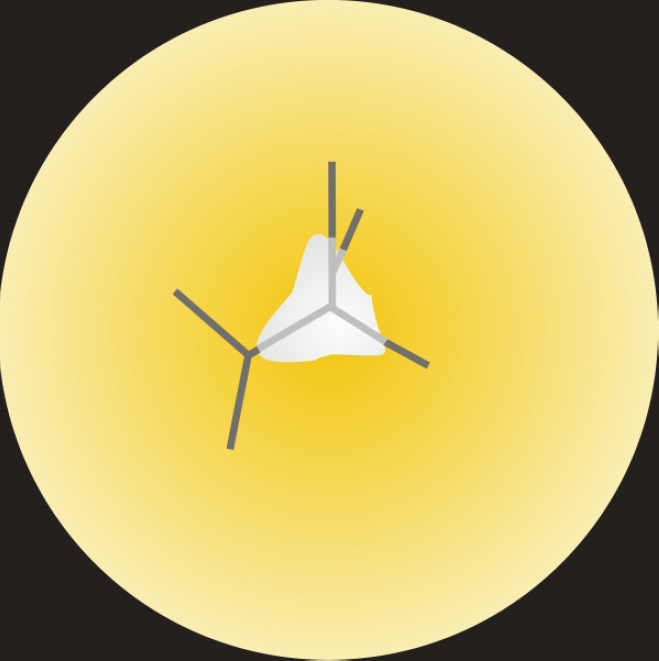
Scaled 3D-CAD animation summarizing the growth, changes in suture pattern and opacity formation in the RCS/Lav rat lenses. The animation begins with the normal posterior Y suture pattern at 2 weeks postnatal and follows the changes through 7–8 weeks postnatal, when the PSC completely obscures the suture. The changes in suture pattern begin as early as 3 weeks postnatal and continue to be altered with age. The loss of transparency is first seen as a sutural opacity at around 5 weeks of age, progressing to a large PSC by 7–8 weeks postnatal. Note that the slide bar at the bottom of the quicktime movie can be used to manually control the flow of the movie. If you are unable to view the movie, a representative frame for each movie is included below.

Changes in posterior suture patterns and transparency were assessed using photographs of the posterior surface of unfixed RCS rat lenses immediately following sacrifice. The observations reveal that RCS rat lenses demonstrated rapid, abnormal changes from the expected suture pattern. At 2 weeks post natal, all lenses presented with the normal ‘inverted Y’ suture on the posterior surface (Figure 2A), however this pattern began to show changes in some lenses as early as 3 weeks postnatal, with the earliest change being the formation of one or more small sub-branches. Suture pattern alterations were more evident by 4 weeks postnatal with the formation of multiple abnormal suture sub-branches (Figure 2B, arrows). By 5 weeks, a sutural opacity was evident in most lenses (Figure 2C, arrowhead) and the opacity completely circumscribed the extent of the posterior suture branches (Figure 2D, arrows). The sutural opacity continued to expand between 5 and 6 weeks post natal (Figure 2E) and completely obscured the posterior suture by about 7–8 weeks post natal (Figure 2F). The genetically matched control lenses (RCS-rdy^+^/Lav) showed the expected inverted Y suture pattern at all age groups (Figure 2G-I). A scaled 3D-CAD animation (Figure 3) summarizes the changes (detailed above) in lens posterior suture pattern and transparency that occur in the dystrophic RCS rat lens between 2 and 8 weeks of age. The animation shows the alterations that begin as early 3 weeks of age and progresses gradually through the concomitant changes in suture pattern and the loss of transparency, with eventual PSC formation in the RCS rat lens. The growth of the lens is also depicted with phases of slower or paused growth that is seen by 7–8 weeks postnatal. By this age, the opacity continued to expand in size, but the lens by itself did not show a significant change in the equatorial diameter.

### Lens posterior fiber ends (SEM)

SEM imaging was performed on the posterior surface of RCS rat lenses that were decapsulated to expose the posterior fiber ends. The posterior aspect of the lens was imaged to visualize fiber end morphology, fiber end arrangement, filopodial structure and filopodial organization. Filopodia are thin actin-rich protrusions of the cell membrane present at the leading edge of migrating cells, and are used by cells as ‘molecular antennae' to probe their micro-environment [17]. Similar to other migratory cells, filopodial extensions have been documented in the migrating fiber ends in the lens as well [18]. An assessment of the filopodial shape, size, arrangement and direction over time, was used to estimate normal and deviant migration of fiber ends toward their sutural destinations.

**Figure 4:**
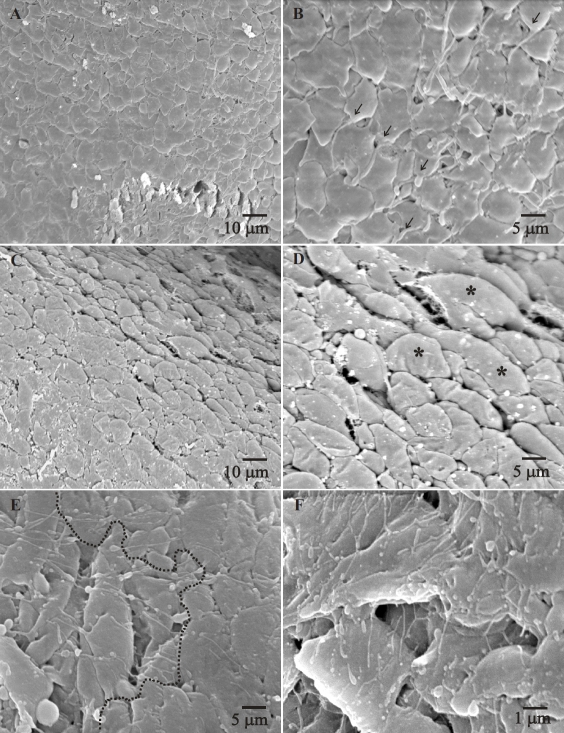
Scanning electron micrographs of RCS/Lav lenses showing the morphology and arrangement of the posterior fiber ends (2–6 weeks). **A**, **B**: Low and high magnification images showing the normal shape and arrangement of the basal ends at 2 weeks of age. Note the uniform size and orientation of the filopodia in panel **B** (arrows). **C**, **D**: Low and high magnification images at 3 weeks postnatal, showing some areas with domed ends (asterisks in panel **D**). For the most part, the ends continue to maintain their normal shape and arrangement as seen at 2 weeks of age. **E**, **F**: By 4 weeks postnatal, fiber ends show distinct changes in their shape, end to end adhesion and the filopodia. Panel **E** shows the border between ends within the forming opacity (left of the dotted line) and normal ends just outside the opacity. The disordered and lengthened filopodia, membrane blebbing and loss of end to end adhesion can be clearly seen in Panel **F**.

**Figure 5:**
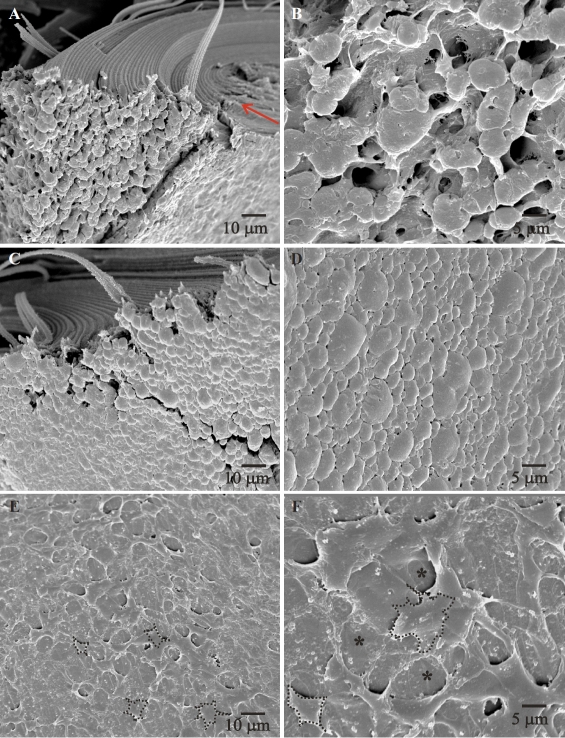
Scanning electron micrographs of RCS/Lav lenses showing the morphology and arrangement of the posterior fiber ends (7–8 weeks). **A**, **B**: Low and high magnification view of the PSC plaque. The arrow points to the original suture plane which is eventually obscured by the PSC, as a result of a cessation of fiber ends migration. The ends within the plaque are dilated/globular with loss of end to end interaction. **C**, **D**: Some PSC plaques maintained the end to end interaction, but were severely dilated and had completely lost their filopodia. Panel **D** also clearly depicts the variability in fiber end size that is seen at this age. **E**, **F**: Some areas which were more severely affected showed stellate shaped ends with surrounding areas of apparent fiber end loss (panel **F**-asterisks).

**Figure 6:**
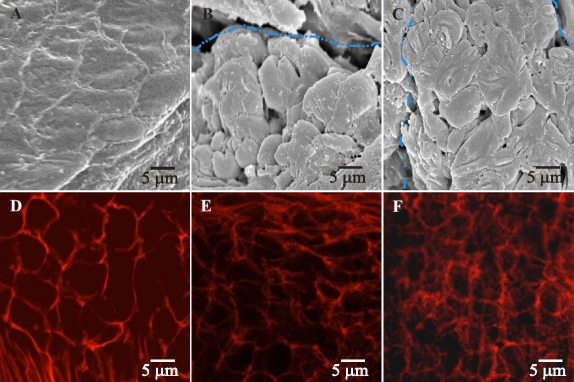
Scanning electron micrographs and LSCM images of RCS-rdy^+^/Lav lenses (controls). A, B, C: SEM images of control lenses at 2–3, 4–6 and 7–8 weeks postnatal respectively showed a normal size, shape and arrangement of posterior fiber ends. Filopodia can be seen extending uniformly in the direction of fiber end migration. Panels B and C show fiber ends in the sutural domain with blue dotted lines indicating the position of suture branches. The ends in this area exhibit round profiles and is consistent with the expected fiber end morphology within sutural domains. D, E, F: Confocal images of control lenses at 2–3, 4–6 and 7–8 weeks postnatal showing the distribution of F-actin. As expected, F-actin clearly outlines the fiber end profiles and lateral membranes at all age groups.

At 2–3 weeks postnatal, posterior fiber ends showed a normal size, shape and packing, i.e., the ends were irregularly spheroidal in shape, tightly packed and exhibited small filopodia in the direction of fiber end migration (Figure 4A,B, arrows indicate filopodial uniformity). Some lenses at 3 weeks showed scattered areas where the ends had a ‘domed’ shape (Figure 4C,D) but overall the filopodia continued to be arranged unidirectionally toward the posterior suture. At 4 weeks postnatal, the organized arrangement of the fiber ends was lost. Notable changes included: disorganized arrangement of the posterior fiber ends with a loss of tight packing between fiber ends (extracellular space [ECS] dilations), elongated filopodia, filopodial tips taking on a ‘bouton-like’ appearance and random orientation of filopodia (Figure 4E,F). Figure 4E clearly shows the border (dotted line) between the normal arrangement (right) and the abnormal fiber ends situated within the confines of the PSC plaque (left). The majority of the changes observed took place between 4 and 6 weeks postnatal and corresponded with PSC formation and suture pattern changes that were observed in the gross lens at the same age. By 7–8 weeks postnatal, the PSC was fully formed and the fiber end segments exhibited the characteristic change of direction away from the polar axis toward the capsule (Figure 5A,C) as has been previously described [14,15]. SEM images of the fiber ends within the PSC plaque at 7–8 weeks postnatal revealed two distinct sets of features; the ends either exhibited ECS dilations, altered morphology and disordered filopodia (Figure 5A,B) or they assumed a distended or globular appearance in a tightly packed configuration with complete lack of filopodia (Figure 5C,D). A subset of the lenses (less than 1/4) showed discrete areas wherein fiber ends had a stellate appearance with an apparent degree of fiber end loss (Figure 5E,F). RCS lenses of all ages in the control group exhibited fiber end shape, size and arrangement consistent with that seen in other rodent strains (Figure 6A-C), specifically, the fiber ends were uniform in size shape and exhibited a normal filopodial anatomy and organization. Fiber end morphology of the 2 week old dystrophic lenses, were comparable to the controls.

### F-Actin Distribution (LSCM)

**Figure 7:**
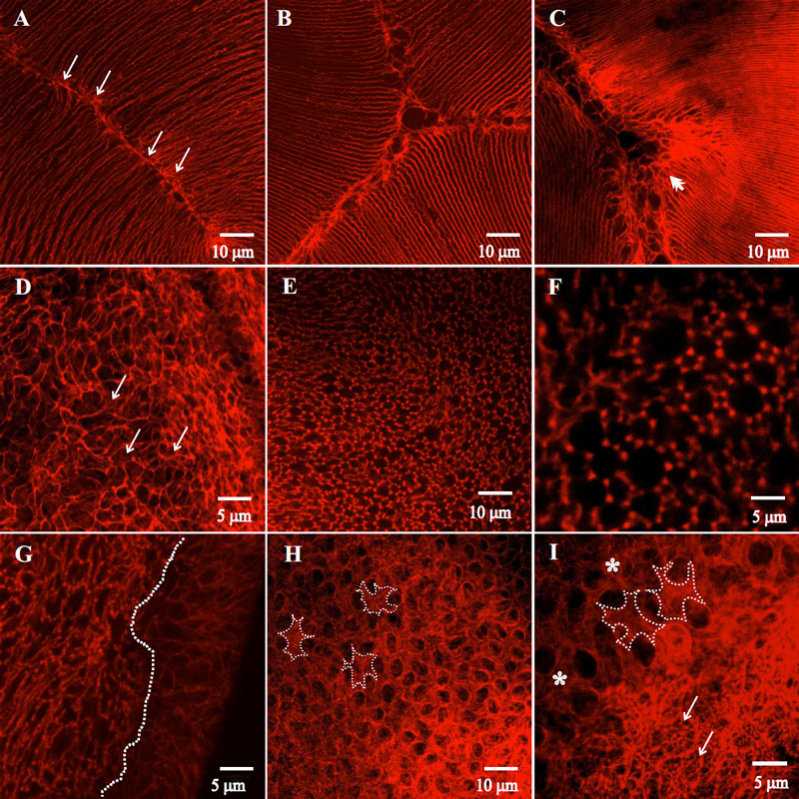
LSCM images showing the F-actin distribution in RCS/Lav rat lenses. **A**, **B**, **C**: F-actin in posterior fiber segments approaching sutures at 2–3 weeks (**A**), 4–6 weeks (**B**) and 7–8 weeks (**C**) clearly outlined the lateral membranes at all ages. A few scattered foci of actin were seen (arrows); panel **C** clearly shows a forming suture sub-branch (double arrowhead). **D**: F-actin delineated the fiber ends at 2–3 weeks and discrete scattered foci were visualized (arrows). **E**, **F**: Low and high magnification images showing the distinct rearrangement of F-actin into ‘rosettes’ at the vertices by 4–6 weeks. **G**: The dotted line depicts the border of the PSC plaque with fiber ends within the plaque (left of the border) showing the F-actin foci while the ends outside the confines of the plaque do not show the F-actin rearrangement. **H**, **I**: Low and high magnification images showing the drastic change in shape of the fiber ends in a subset of severely affected lenses. The ends were stellate shaped (panel **I**, asterisks) with surrounding areas of degeneration. Some fiber ends showed a fibrillar actin network present within them (panel **I**, arrows).

Correlative confocal microscopy was used to visualize the distribution of F-actin in the lateral fiber membranes and BMC of fiber ends during PSC formation. In the control lenses at all age groups, F-actin was found to delineate the fiber ends clearly and was also present along the lateral membranes (Figure 6D-F). In the dystrophic lenses, F-actin was present along the lateral membranes at all ages (Figure 7A-C) and clearly delineated the periphery of the BMC in fiber ends at 2 – 3 weeks postnatal (Figure 7D, arrows), consistent with the controls. Between 4 and 6 weeks postnatal, (corresponding to the structural changes seen by SEM), F-actin in the BMC was reorganized such that it was arranged in a ‘rosette’ pattern around individual fiber end profiles. Fiber ends displayed from 4 – 9 foci which appeared to be located at the vertices (Figure 7E,F). Visualization of the PSC showed that the F-actin foci were present exclusively within the confines of the plaque (Figure 7G, the PSC plaque is to the left of the dotted line**)**. By 7–8 weeks postnatal, F-actin distribution reflected the structural damage seen by SEM and revealed areas of fiber end loss and abnormal stellate shape of the ends (Figure 7H,I, asterisks). An F-actin network was visible within some of the posterior fiber ends (Figure 7I, arrows). The dilated ends seen in some of the PSC plaques (Figure 5D) displayed faint F-actin fluorescence with scattered foci (data not shown).

**Figure 8:**
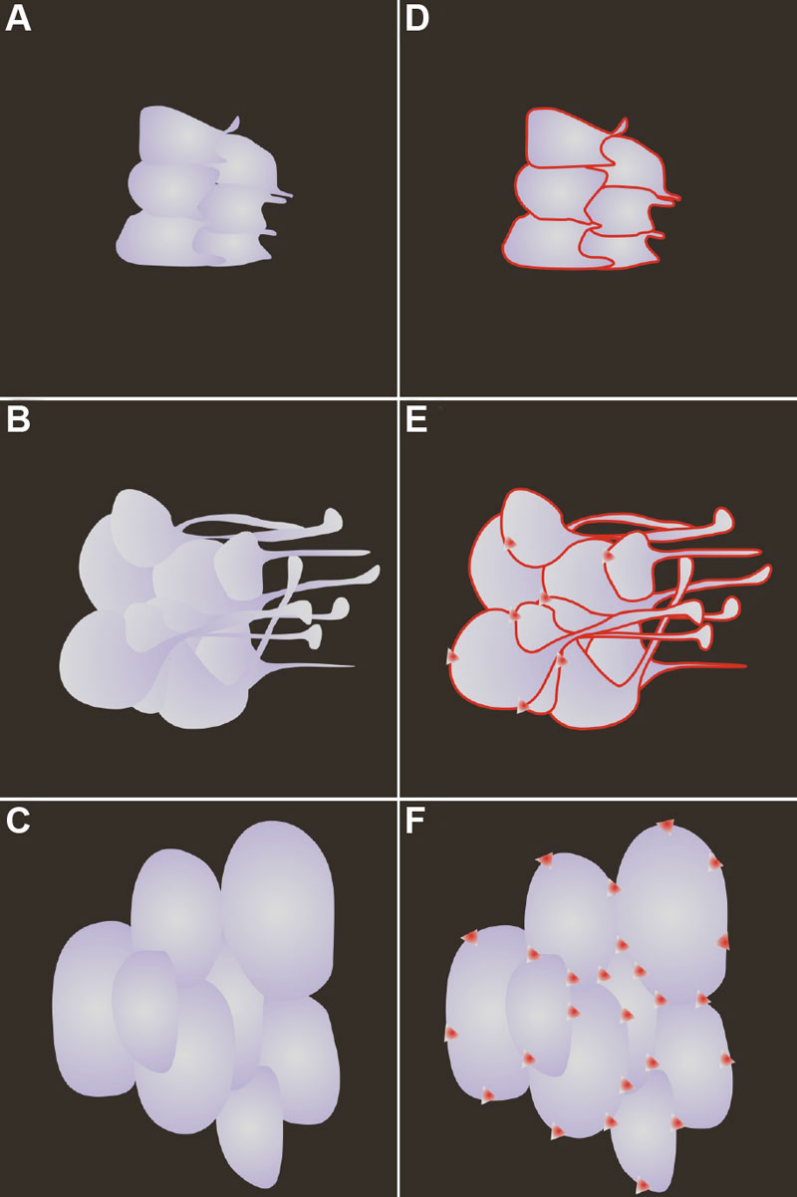
Schematic representation of the posterior fiber ends of RCS/Lav rat lenses depicting the change in fiber end morphology, filopodial configuration and F-actin distribution as a function of time. **A**-**C**: Note the change in fiber end shape from irregular spheroids having short, unidirectional filopodia at 2-3 weeks of age to enlarged, disorganized ends with elongated filopodia at 4–6 weeks. By 7–8 weeks, the ends were extremely dilated and globular with complete absence of filopodia. The concomitant F-actin rearrangements at the corresponding ages are also diagrammatically summarized in panels **D**, **E**, and **F**. At 2–3 weeks of age, F-actin distribution was predominantly peripheral within the BMC (**D**), however by 4–6 weeks, foci were common (**E**) within the forming PSC plaque. The F-actin was entirely rearranged into rosettes within the dilated fiber ends (**F**).

The sequential transition in fiber end morphology, specifically the altered shape, filopodial configuration and end – end relationships at the posterior fiber ends along with the F-actin configuration is depicted diagrammatically in Figure 8.

## Discussion

**Figure 9:**
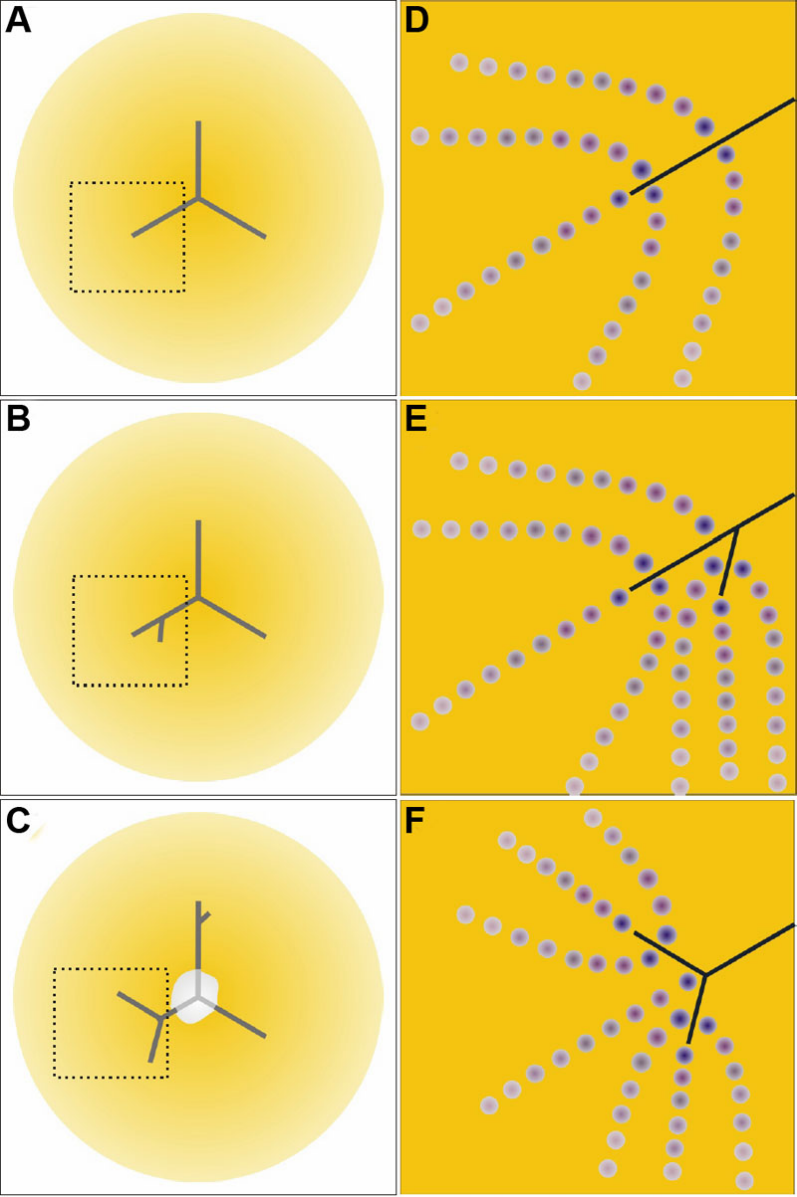
Schematic model depicting the rapid shift in migration patterns of posterior fiber ends during the initial stages of PSC formation in RCS/Lav rat lenses. **A**, **D**: Initially, the posterior suture pattern (gray) is a three branched inverted Y (**A**), wherein fiber ends (**D**; gradations of purple) migrate either along meridians to the proximal ends of the suture branch or along a curved path to the lateral aspect of the suture branch. Successive cohorts of migrating fiber ends are depicted in decreasing intensities of color, with the oldest generation denoted by the darkest color. **B**, **E**: The first noticeable change is the rapid formation of abnormal suture sub-branches (**B**), due to the change in migration paths of selected groups of fiber ends (**E**). **C**, **F**: Over time, the elaboration of abnormal sub-branches continues (**F**) as migration paths undergo additional shifts through successive cohorts of fiber ends.

This study documented the structural changes that are seen at the posterior fiber ends of RCS rat lenses during the process of PSC formation in these animals. Up to 6 weeks of age, the lenses grew in size while concomitantly showing the deleterious effects resulting from retinal dystrophy, namely, the generation of abnormal suture patterns and opacity (PSC) formation. The changes in suture pattern included the formation of one or two suture sub-branches as early as 3 weeks postnatal and the addition of more sub-branches by 4 weeks. These changes in suture pattern were extremely rapid and were noticeable well before the PSC could be observed. In a previous study, we documented the rapid formation of such abnormal suture sub-branches in Wistar rats [19] and have shown this process to be a consequence of pathology, distinctly different from secondary suture sub-branch formation during the normal aging process [20,21]. It follows that the normal migratory paths of basal fiber ends were perturbed, resulting in the brisk development of suture sub-branches in the RCS rat lens. This process has been diagrammatically depicted in Figure 9. As the diversity of the suture pattern increased, the transparency of the lens was also affected. The PSC in the extracted RCS lenses formed as a ‘granular plaque’ and was situated along the visual axis. This presentation closely resembles prior descriptions of PSC plaques from varying etiologies, including ‘granular/lace-like’ and ‘sugar-grain’ [5,13,22]. The axial position of the PSC plaque has an extremely deleterious impact on vision, and the characteristics of the opacification indicate a resultant increase in light scatter as well. It is well known that major structural alterations seen in various forms of cataracts result in increased light scatter and a consequent loss of transparency [23-27]. It follows therefore that the structural changes, deviant fiber end migration and altered suture patterns that are observed and documented in the present model would also result in increased light scatter and loss of lens transparency.

The initiation of PSC formation begins with a small sutural or central opacity being observed as early as 5 weeks postnatal. The opacity continued to grow in dimensions until it circumscribed the extent of the posterior suture branches by about 6 weeks postnatal and completely obscured the posterior suture by 7–8 weeks postnatal (Figure 2F). Interestingly, by about this time the equatorial diameter of the lens remained static suggesting that once the PSC was formed, all fiber end migration, both normal and misdirected, temporarily ceased. Prior studies have shown that lens growth resumes after 10–12 weeks postnatal and eventually internalizes the PSCs [15,16].

As expected, the evaluation of posterior fiber ends revealed distinct morphological changes during cataractogenesis, specifically, the shape, end to end packing, organized arrangement, and the filopodial configuration and direction showed a marked variation from normal. The first noticeable change at 3 weeks postnatal was the altered shape of the fiber ends. The irregularly spheroidal outlines were occasionally interspersed with areas characterized by ‘domed’ ends. Although these ends exhibited an appreciable difference in their morphology, they continued to maintain contact with the surrounding ends, presumably via adherens junctions. In fact, prior investigations have shown that the basal membrane complex (BMC) has ample cadherin which mediates interactions within and between cohorts of migrating fiber ends in normal lenses [28,29]. In 4 week old RCS rat lenses, this interaction between fiber ends was disrupted (Figure 4E,F), resulting in areas of ECS dilations and end separation, indicating that end to end adhesion was partially lost. The initial structural changes began by 4 weeks of age and corresponded temporally to the variations seen in the posterior suture pattern of the RCS rat lens. One possible mechanism for the maintenance of coordinated fiber end migration suggests that the fiber ends in each successive generation simply ‘follow-the-lead’ of the prior cohort of fiber ends as they travel to their sutural destinations. If this is correct, even partial disruption of cell to cell adhesion could contribute to the aberrant migration pattern in the present model. It is clear that future investigations must address the issue of cell to cell adhesion during PSC formation in the RCS rat lens.

It is known that the leading edge of a migrating cell is a site of rapid actin polymerization forming membrane extensions in the form of lamellipodia or filopodia that play a crucial role in directing the cell during migration [30-32]. Likewise, the uniform arrangement of filopodia at the posterior fiber ends in the lens has been a good indicator of the orderly and directed end migration toward the posterior suture [18]. Conversely, the presence of disordered filopodia has been used in prior studies as a gauge to assess aberrant lens fiber end migration and/or abnormal fiber end – BMC interaction [19,33]. In the RCS rat, although the filopodial extensions at 2 to 3 weeks postnatal were uniformly aligned toward the sutures, they were distinctly different in their morphology and alignment by 4 to 6 weeks of age. Specifically, they exhibited excessive lengthening, the formation of ‘bouton-like’ dilations at their tips and the loss of directionality toward the posterior suture. The severely altered filopodial configurations in RCS rat lens fiber ends seem to point out a drastic misdirection of the migrating posterior fiber ends, and this correlates with the changes seen in the suture pattern at the corresponding ages, namely the rapid formation of multiple suture sub-branches and the incipient PSC. It is to be noted that at this age, although there were areas where fiber ends had separated, most groups of fiber ends continued to migrate en-masse, albeit along aberrant migratory paths that resulted in the abnormal suture sub-branches. By 7–8 weeks postnatal, there were areas of fiber ends that exhibited either a stellate appearance, or areas with dilated ends of variable sizes. Ends that showed the stellate shape were almost always situated in areas corresponding to severe damage, as evidenced by the degeneration of surrounding fiber ends. Both these morphologies could indicate a complete cessation of migration, as indicated by the lack of new suture sub-branch formation and the static equatorial diameter of the lens at this age. Examination of the fully formed PSC also revealed the characteristic turning of the affected fibers toward the capsule thus forming new suture planes, as has been previously described in this rodent model [14,15].

Cellular morphology is often dictated by the cytoskeleton, specifically actin configurations. To better understand the morphological changes that were observed in the RCS rat, cytochemical techniques were used to elucidate F-actin distribution in the BMC of the posterior fiber ends. As expected, F-actin arrangement in lenses up to 3 weeks postnatal was comparable to normal distributions seen in other rodent strains, and the outlines very succinctly revealed the normal shape, size and arrangement of the fiber ends, concomitant to that seen in the SEM data. By 4–6 weeks postnatal, F-actin underwent a very unique redistribution and was no longer present as an unbroken outline around the fiber ends; rather bright foci of actin fluorescence were visible at fiber end vertices in a distinctive ‘rosette’ arrangement (Figure 7E,F). The relatively normal shape of the ends could still be visualized through the interrupted F-actin outline, indicating that this rearrangement could transiently help in maintaining the shape and end – end contact, even as the filopodial configurations resulted in misdirected fiber end migration. With increasing age, the damage seen via SEM imaging was reflected in the F-actin distribution with areas of fiber end loss and the stellate shape of affected fiber ends.

Elongating lens fibers and migrating fiber ends typically show an increased association of F-actin at the membranes [34,35], with fiber ends being delineated clearly [18]. In the RCS rat lens, fiber end migration is affected and the distinct rearrangement of F-actin could be a possible explanation for this transient cessation of migration. The foci of F-actin could be behaving as ‘molecular anchors’ that hold the ends down, preventing their fluid movement across the posterior capsule and possibly altering the fiber end – BMC interactions. Although the basis for this rearrangement requires further study, we speculate that lens pathology could be the result of an inflammatory response to factors released during the retinal degeneration in the RCS rat. Cytokine induced actin depolymerization is known to occur in several biologic systems [36,37] resulting in altered cell shape, migratory potential and F-actin rearrangements [38]. Preliminary investigations in our laboratory point toward pro-inflammatory cytokines playing a role during cataractogenesis in the RCS rat. In an alternate model of PSC formation, inflammatory responses have been shown to affect lens structure [39], and anti-inflammatory agents have been successfully used to prevent the PSC formation [40,41]. Thus, actin rearrangement could be a contributing factor in PSC formation in the present model.

Another possible interpretation is that the rearrangement of F-actin occurs as a compensatory mechanism that prevents fiber end degeneration during PSC formation. As observed within the cataract plaque, the fiber ends revealed a dilated appearance (Figure 5C,D) with discrete foci at vertices (Figure 7F), which could indicate a rearrangement of F-actin as ‘tensegrity structures’. Tensegrity is an architectural system in which the tensile and compressive forces are balanced thus rendering stability [42,43] as can be seen in buckminsterfullerene structures or geodesic domes. This concept has several applications in biology [44-46]; in the human body, the musculo-skeletal system and the central nervous system behave as tensegrities and at the cellular and molecular level, the cytoskeleton is a prime example of a tensegral system [47,48]. We speculate that the rapid alteration of fiber end migration paths results in the F-actin being reorganized into configurations that help support and maintain the shape of the fiber ends, thus preventing their complete breakdown. Similar to the concept of tensegrity in architecture, to balance the combined compressive and tensile forces that could potentially be acting on these fiber ends during phases of misdirected and/or uncontrolled migration, the F-actin could be rearranging itself into a structure that is tensegral. It is possible that the ‘rosette’ arrangement of F-actin seen during the initial PSC formation closely mimics a tensegral structure, rendering some semblance of stability to a rapidly deteriorating system. This would be especially advantageous in the fiber ends that are excessively enlarged and domed and could be the initial step that leads to the internalization and recovery of the lens previously documented in this animal model [15,16]. Our studies indicate that lenses that develop dilated fiber ends stabilized by F-actin adopting tensegral configurations go on to recover by internalizing the PSC. In contrast, those lenses wherein the ends become stellate and are associated with fiber end degeneration (Figure 5E,F) ultimately progress to complete cortical liquefaction [49]. The above indicates that the molecular reconfiguration of F-actin seems to play an important role in preventing the degeneration of cortical fibers, thus facilitating the lens recovery process.

In conclusion, this study elucidated the structural changes that affect migrating fiber ends in the RCS rat during the initiation and development of a posterior subcapsular cataract. The rapid alteration of lens fiber end migration leads to abnormal suture sub-branch formation with a concomitant change in fiber end morphology and F-actin configuration, ultimately resulting in PSC formation. Continued studies in our laboratory are presently focused on elucidating other BMC molecules responsible for these structural changes and agents that could potentially be responsible for these distinct alterations.

## Supplementary Material

Supporting Movie

## References

[1] Bourne MC Campbell DA Tansley K Hereditary degeneration of the rat retina. Br J Ophthalmol 1938 22 613 23 18169569 10.1136/bjo.22.10.613 PMC1142980

[2] Bourne MC Gruneberg H Degeneration of the Retina and Cataract- A new recessive gene in the rat (Rattus norvegicus). J Hered 1939 30 131 6

[3] LaVail MM Sidman RL Gerhart CO Congenic strains of RCS rats with inherited retinal dystrophy. J Hered 1975 66 242 4 1172515 10.1093/oxfordjournals.jhered.a108621

[4] Bourne MC Campbell DA Pyke M Cataract associated with an hereditary retinal lesion in rats. Br J Ophthalmol 1938 22 608 13 18169568 10.1136/bjo.22.10.608 PMC1142979

[5] Hess HH Newsome DA Knapka KK Westney GE Slitlamp assessment of age of onset and incidence of cataracts in pink-eyed, tan-hooded retinal dystrophic rats. Curr Eye Res 1982-1983 2 265 9 7151470 10.3109/02713688209011629

[6] Berman ER Merin S Segal N Photiou S Approaches to human retinitis pigmentosa through studies of vitamin A metabolism in the RCS (dystrophic) rat. Birth Defects Orig Artic Ser 1982 18 135 48 7171749

[7] Hess HH Newsome DA Knapka JJ Westney GE Slit-lamp assessment of onset of cataracts in black-eyed, black-hooded retinal dystrophic rats. Invest Ophthalmol Vis Sci 1983 24 654 7 6841017

[8] Gal A Li Y Thompson DA Weir J Orth U Jacobson SG Apfelstedt-Sylla E Vollrath D Mutations in MERTK, the human orthologue of the RCS rat retinal dystrophy gene, cause retinitis pigmentosa. Nat Genet 2000 26 270 1 11062461 10.1038/81555

[9] Dowling JE Sidman RL Inherited retinal dystrophy in the rat. J Cell Biol 1962 14 73 109 13887627 10.1083/jcb.14.1.73 PMC2106090

[10] Mullen RJ La Vail MM Inherited retinal dystrophy: primary defect in pigment epithelium determined with experimental rat chimera. 1976 192 799 801 1265483 10.1126/science.1265483

[11] Herron WL Riegel BW Myers OE Rubin ML Retinal dystrophy in the rat - a pigment epithelial disease. Invest Ophthalmol Vis Sci 1969 8 595 604 5359576

[12] Zigler JS Jr Bodaness RS Gery I Kinoshita JH Effects of lipid peroxidation products on the rat lens in organ culture: a possible mechanism of cataract initiation in retinal degenerative disease. Arch Biochem Biophys 1983 225 149 56 6614915 10.1016/0003-9861(83)90018-8

[13] Zigler JSJ Hess HH Cataracts in the Royal College of Surgeons rats. Evidence for initiation by lipid peroxidation products. Exp Eye Res 1985 41 67 76 4029287 10.1016/0014-4835(85)90095-8

[14] Al-Ghoul KJ Novak LA Kuszak JR The structure of posterior subcapsular cataracts (PSCs) in the Royal College of Surgeons (RCS) rats. Exp Eye Res 1998 67 163 77 9733583 10.1006/exer.1998.0505

[15] Al-Ghoul KJ Novak LA Peterson KL Kuszak JR The internalization of posterior subcapsular cataracts (PSCs) in Royal College of Surgeons (RCS) rats. I. Morphological characterization. Mol Vis 1999 5 6 10329769

[16] Kuszak JR Al-Ghoul KJ Novak LA Peterson.K.L., Herbert KL, Sivak JG. The internalization of posterior subcapsular cataracts (PSCs) in Royal College of Surgeons (RCS) rats. II. The inter-relationship of optical quality and structure as a function of age. Mol Vis 1999 5 7 10329770

[17] Mattila PK Lappalainen P Filopodia: molecular architecture and cellular functions. Nat Rev Mol Cell Biol 2008 9 446 54 18464790 10.1038/nrm2406

[18] Al-Ghoul KJ Kuszak JR Lu JY Owens MJ Morphology and organization of posterior fiber ends during migration. Mol Vis 2003 9 119 28 12707642

[19] Joy A Currie MS Donohue ST Al Ghoul KJ Aberrant basal fiber end migration underlies structural malformations in a streptozotocin-induced diabetic rat model. Exp Eye Res 2009 89 344 57 19358842 10.1016/j.exer.2009.03.022 PMC2720437

[20] Kuszak JR Zoltoski RK Tiedemann CE Development of lens sutures. Int J Dev Biol 2004 48 889 902 15558480 10.1387/ijdb.041880jk

[21] Donohue ST, Al-Ghoul KJ. The Initiation Of Suture Branch Formation In Rat Lenses. ARVO Annual Meeting; 2008 April 26-May 1; Fort Lauderdale (FL).

[22] Brown NAP, Bron AJ. Lens disorders. A clinical manual of cataract diagnosis. Oxford, UK: Butterworth-Heinemann Ltd.; 1996.

[23] Tripathi RC Tripathi BJ Lens morphology, aging, and cataract. J Gerontol 1983 38 258 70 6841920 10.1093/geronj/38.3.258

[24] Clark JI Mengel L Bagg A Benedek GB Cortical opacity, calcium concentration and fiber membrane structure in the calf lens. Exp Eye Res 1980 31 399 410 7449875 10.1016/s0014-4835(80)80024-8

[25] Harding CV, Chylack LT, Susan SR, Decker JG, Lo WK. Morphological changes in the cataract: the ultrastructure of human lens opacities, localized by Cooperative Cataract Research Group procedures. In: Srivastava SK, editors. Red blood cells and lens metabolism. New York: Elsevier/North Holland Inc.; 1980. p. 27–39.

[26] Vrensen G Willekens B Biomicroscopy and scanning electron microscopy of early opacities in the aging human lens. Invest Ophthalmol Vis Sci 1990 31 1582 91 2387688

[27] Obazawa H Electron microscopic studies on the lens nucleus in cataract. I. Normal and senile cataractous lens. Nippon Ganka Gakkai Zasshi 1967 71 1019 28 5625236

[28] Bassnett S Missey H Vucemilo I Molecular architecture of the lens fiber cell basal membrane complex. J Cell Sci 1999 112 2155 65 10362545 10.1242/jcs.112.13.2155

[29] Lu JY Mohammed TA Donohue ST Al Ghoul KJ Distribution of basal membrane complex components in elongating lens fibers. Mol Vis 2008 14 1187 203 18596883 PMC2442472

[30] Cramer LP Mitchison TJ Theriot JA Actin-dependent motile forces and cell motility. Curr Opin Cell Biol 1994 6 82 6 8167030 10.1016/0955-0674(94)90120-1

[31] Mitchison TJ Cramer LP Actin-based cell motility and cell locomotion. Cell 1996 84 371 9 8608590 10.1016/s0092-8674(00)81281-7

[32] Cohan CS Welnhofer EA Zhao L Matsumura F Yamashiro S Role of the actin bundling protein fascin in growth cone morphogenesis: localization in filopodia and lamellipodia. Cell Motil Cytoskeleton 2001 48 109 20 11169763 10.1002/1097-0169(200102)48:2<109::AID-CM1002>3.0.CO;2-G

[33] Norose K Lo WK Clark JI Sage EH Howe CC Lenses of SPARC-null mice exhibit an abnormal cell surface-basement membrane interface. Exp Eye Res 2000 71 295 307 10973738 10.1006/exer.2000.0884

[34] Lee A Fischer RS Fowler VM Stabilization and remodeling of the membrane skeleton during lens fiber cell differentiation and maturation. Dev Dyn 2000 217 257 70 10741420 10.1002/(SICI)1097-0177(200003)217:3<257::AID-DVDY4>3.0.CO;2-5

[35] Sue Menko A Lens epithelial cell differentiation. Exp Eye Res 2002 75 485 90 12457861 10.1006/exer.2002.2057

[36] Bonventre JV Weinberg JM Recent advances in the pathophysiology of ischemic acute renal failure. J Am Soc Nephrol 2003 14 2199 210 12874476 10.1097/01.asn.0000079785.13922.f6

[37] Molitoris BA Actin cytoskeleton in ischemic acute renal failure. Kidney Int 2004 66 871 83 15253754 10.1111/j.1523-1755.2004.00818.x

[38] Kutsuna H Suzuki K Kamata N Kato T Hato F Mizuno K Kobayashi H Ishii M Kitagawa S Actin reorganization and morphological changes in human neutrophils stimulated by TNF, GM-CSF, and G-CSF: the role of MAP kinases. Am J Physiol Cell Physiol 2004 286 C55 64 12954601 10.1152/ajpcell.00131.2003

[39] Kuszak JR Sivak JG Herbert KL Scheib S Garner WH Graff G The relationship between rabbit lens optical quality and sutural anatomy after vitrectomy. Exp Eye Res 2000 71 267 81 10973736 10.1006/exer.2000.0877

[40] Kuszak JR Sivak JG Herbert KL Scheib S Garner W Graff G Inhibition of vitrectomy induced cataracts in rabbits with the use of a novel AL-8417 irrigating solution. Invest Ophthalmol Vis Sci 1999 40 s197

[41] Kuszak JR Sivak JG Moran KL Scheib SA Garner WH Ke TL Hellberg MR Graff G Suppression of post-vitrectomy lens changes in the rabbit by novel benzopyranyl esters and amides. Exp Eye Res 2002 75 459 73 12387793

[42] Ingber DE The architecture of life. Sci Am 1998 278 48 57 11536845 10.1038/scientificamerican0198-48

[43] Ingber DE Dike L Hansen L Karp S Liley H Maniotis A McNamee H Mooney D Plopper G Sims J Wang N Cellular tensegrity: exploring how mechanical changes in the cytoskeleton regulate cell growth, migration, and tissue pattern during. Int Rev Cytol 1994 150 173 224 8169080 10.1016/s0074-7696(08)61542-9

[44] Stamenović D Fredberg JJ Wang N Butler JP Ingber DE A microstructural approach to cytoskeletal mechanics based on tensegrity. J Theor Biol 1996 181 125 36 8935591 10.1006/jtbi.1996.0120

[45] Wang N Ingber DE Control of cytoskeletal mechanics by extracellular matrix, cell shape, and mechanical tension. Biophys J 1994 66 2181 9 8075352 10.1016/S0006-3495(94)81014-8 PMC1275944

[46] Wang N Naruse K Stamenović D Fredberg JJ Mijailovich SM Tolić-Nørrelykke IM Polte T Mannix R Ingber DE Mechanical behavior in living cells consistent with the tensegrity model. Proc Natl Acad Sci USA 2001 98 7765 70 11438729 10.1073/pnas.141199598 PMC35416

[47] Ingber DE Cellular tensegrity: defining new rules of biological design that govern the cytoskeleton. J Cell Sci 1993 104 613 27 8314865 10.1242/jcs.104.3.613

[48] Ingber DE Mechanical control of tissue morphogenesis during embryological development. Int J Dev Biol 2006 50 255 66 16479493 10.1387/ijdb.052044di

[49] Al-Ghoul KJ Kuszak JR Anterior polar cataracts in RCS rats: A predictor of mature cataract formation. Invest Ophthalmol Vis Sci 1999 40 668 79 10067970

